# Evaluation of the cardioprotective potential of extracellular vesicles – a systematic review and meta-analysis

**DOI:** 10.1038/s41598-018-33862-5

**Published:** 2018-10-24

**Authors:** Sebastian Wendt, Andreas Goetzenich, Claudia Goettsch, Christian Stoppe, Christian Bleilevens, Sandra Kraemer, Carina Benstoem

**Affiliations:** 1 Department of Thoracic and Cardiovascular Surgery, Medical Faculty RWTH Aachen, Aachen, Germany; 2 Cardiovascular Critical Care & Anaesthesia research and evaluation (3CARE), Medical Faculty RWTH Aachen, Aachen, Germany; 3 Department of Internal Medicine I-Cardiology, Medical Faculty RWTH Aachen, Aachen, Germany; 4 Department of Intensive Care Medicine, Medical Faculty RWTH Aachen, Aachen, Germany; 5 Department of Anaesthesiology, Medical Faculty RWTH Aachen, Aachen, Germany

## Abstract

Cardiovascular diseases are the main cause of death worldwide, demanding new treatments and interventions. Recently, extracellular vesicles (EVs) came in focus as important carriers of protective molecules such as miRNAs and proteins which might contribute to e.g. improved cardiac function after myocardial infarction. EVs can be secreted from almost every cell type in the human body and can be transferred via the bloodstream in almost every compartment. To provide an all-encompassing overview of studies investigating these beneficial properties of EVs we performed a systematic review/meta-analysis of studies investigating the cardioprotective characteristics of EVs. Forty-three studies were investigated and catalogued according to the EV source. We provide an in-depth analysis of the purification method, size of the EVs, the conducted experiments to investigate the beneficial properties of EVs as well as the major effector molecule encapsulated in EVs mediating protection. This study provides evidence that EVs from different cell types and body fluids provide cardioprotection in different *in vivo* and *in vitro* studies. A meta-analysis was performed to estimate the underlying effect size. In conclusion, we demonstrated that EVs from different sources might serve as a promising tool for treating cardiovascular diseases in the future.

## Introduction

Cardiovascular diseases, including myocardial infarction (MI), are the main cause of death worldwide^1^. Reduced reperfusion and/or occlusion of the coronary arteries caused e.g. by atherosclerotic plaques results in reduced blood supply of distinct regions of the heart. This in turn, leads to hypoxia and cell death in the myocardium, commonly known as MI demanding an intervention to restore the blood supply of the infarcted region. Controversially, the reperfusion itself causes further damage due to emerging production of reactive oxygen species (ROS) as well as inflammation resulting in ischemia reperfusion (I/R) injury^2^. To counteract these damaging effects, numerous studies investigated the cardioprotective impact and underlying mechanisms of different protective treatments, such as conditioning by ischemia or anaesthetics^3–5^. In this context, extracellular vesicles (EVs) recently gained attention as promising mediators of cardioprotection. EVs are nanometer sized vesicles, which are released by almost every cell in the human body. Exosomes, the smallest group of EVs (30–150 nm), are generated by multiple inward folding of the plasma membrane and are released by fusion of multivesicular bodies (MVBs) with the plasma membrane. Microvesicles are generated through direct budding from the plasma membrane and have a size of 150–1000 nm. The largest type of vesicles are apoptotic bodies with a range of 1–5 µm^6^. It has recently been shown that EVs, especially small EVs (sEVs, exosomes and microvesicles), mediate cardioprotective abilities by transferring cytoprotective proteins and miRNAs^7–9^. For instance, heat shock protein 70 (HSP70) as well as miR-22 can be encapsulated in EVs and trigger pro-survival pathways in the recipient cells to protect those from cell death^7,10^.

We therefore investigated the beneficial effects that EVs might transmit to the heart in a systematic review and meta-analysis conducted in accordance with Cochrane standards. We categorized studies according to EV source, information of the applied EV-purification method, size of the isolated EVs, applied injury model as well as the specific mediator mediating protection inside the EVs. Studies that identified protective EVs were further investigated according to the used methods to identify their protective properties. Finally, a meta-analysis characterized the quality of effect.

## Results

### Study selection process

**Figure 1:**
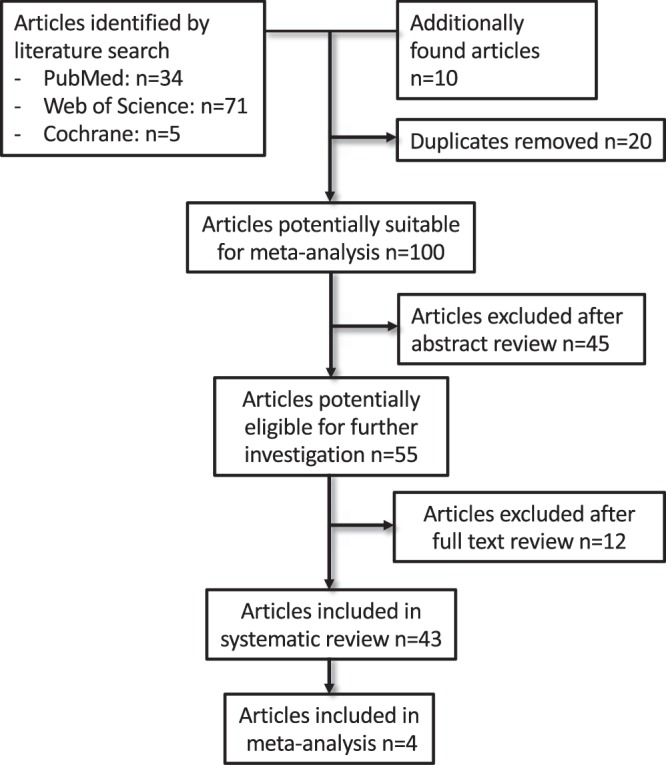
Flow chart of study selection.

Figure 1 represents the process of study selection. According to the search criteria specified in the material and methods section, we identified 110 articles (34 from PubMed, 71 from web of science and 5 from Cochrane). 10 additional articles were found independently from other sources. 20 duplicates were removed. After reviewing title and abstract, we excluded 45 articles, as they did not match our inclusion criteria. We assessed 55 articles during full text screening, 43 were deemed suitable for qualitative analysis and included in this systematic review. Due to heterogeneity throughout the performed experiments in the investigated articles, only four studies were included in the meta-analysis.

### Included studies

The included studies investigated EVs associated with cardioprotection. We first evaluated the quality of EV research by investigating the basic EV-specific experiments (electron microscopy (EM)-images and EV marker). We distinguished the studies by the EV source and extracted which EV purification protocol was used, the EV size, the applied injury model and if applicable the specific mediator inside EVs which mediated the protection. We additionally extracted which experiments were performed to investigate the protective properties of EVs and the investigated EV marker.

### Quality of EV research

The publications were evaluated by basic criteria such as the chosen EV-purification methods, detection of EV related proteins as well the availability of EM pictures. These are our, in accordance with others, minimum criteria which have to be met to ensure an appropriate evaluation if the isolated particles were indeed EVs^11,12^.

The main purification procedures were based on ultracentrifugation^7–10,13–33^ and precipitation methods^10,13,18,21,24,29,34–49^. Six publications used both methods for EV purification^10,13,18,21,24,29^ and five publications used other methods or explicit different additional methods, to isolate EVs^20,29,33,50,51^. Of all investigated articles only nine publications did not perform EM pictures of the isolated EVs^9,19,24,32,40,43,47,50,51^ and three publications did not investigate if the isolated particles contained typical EV markers^19,43,50^. We additionally investigated whether the described methods were reported in the results section (reporting bias) and if any disclosures might have compromised the results (other bias) (Table 1).

**Table 1:** Appraisal of research method (EM images and EV marker) and risk of bias summary.

Publication	EM images	EV marker	Reporting Bias	Other Bias
Arslan *et al*.^50^	−	−	**+**	**+**
Barile *et al*.^13^	**+**	**+**	**+**	**+**
Balbi *et al*.^26^	**+**	**+**	**+**	**+**
Bang *et al*.^17^	**+**	**+**	**+**	**+** ^a^
Shi *et al*.^48^	**+**	**+**	**+**	**+**
Borosch *et al*.^33^	**+**	**+**	**+**	**+**
Chen *et al*.^34^	**+**	**+**	**+**	?
Cheow *et al*.^18^	**+**	**+**	**+**	?
Davidson *et al*.^27^	**+**	**+**	**+**	**+**
De Couto *et al*.^47^	−	**+**	**+**	**+** ^a^
Feng *et al*.^10^	**+**	**+**	**+**	**+**
Garcia *et al*.^21^	**+**	**+**	**+**	**+** ^a^
Giricz *et al*.^8^	**+**	**+**	**+**	**+**
Gray *et al*.^22^	**+**	**+**	**+**	**+**
Gu *et al*.^19^	−	−	**+**	**+**
Ibrahim *et al*.^35^	**+**	**+**	**+**	?
Kang *et al*.^14^	**+**	**+**	**+**	**+**
Kang *et al*.^36^	**+**	**+**	**+**	**+**
Lai *et al*.^20^	**+**	**+**	**+**	?
Li *et al*.^37^	**+**	**+**	**+**	**+** ^a^
Ma *et al*.^15^	**+**	**+**	**+**	**+**
Minghua *et al*.^49^	**+**	**+**	**+**	**+**
Namazi, Mohit *et al*.^28^	**+**	**+**	**+**	**+**
Namazi, Namazi *et al*.^25^	**+**	**+**	**+**	?
Obata *et al*.^29^	**+**	**+**	**+**	**+** ^a^
Ong *et al*.^38^	**+**	**+**	**+**	**+**
Ribeiro-Rodrigues *et al*.^30^	**+**	**+**	**+**	**+**
Svennerholm *et al*.^16^	**+**	**+**	**+**	**+**
Svennerholm *et al*.^31^	**+**	**+**	**+**	**+**
Teng *et al*.^44^	**+**	**+**	**+**	**+**
Vandergriff *et al*.^51^	−	**+**	**+**	**+**
Vicencio *et al*.^7^	**+**	**+**	**+**	**+**
Y. Wang *et al*.^45^	**+**	**+**	**+**	**+**
X. Wang *et al*.^9^	−	**+**	**+**	**+**
X. Wang *et al*.^24^	−	**+**	**+**	**+**
Wider *et al*.^32^	−	**+**	**+**	**+**
Xiao *et al*.^39^	**+**	**+**	**+**	**+**
Yamaguchi *et al*.^40^	−	**+**	**+**	**+**
Yu *et al*.^41^	**+**	**+**	**+**	**+**
Yu *et al*.^42^	**+**	**+**	**+**	**+**
Zhang *et al*.^46^	**+**	**+**	**+**	**+**
Zhang *et al*.^43^	−	−	**+**	**+**
Zhao *et al*.^23^	**+**	**+**	**+**	**+**

^+^Indicates that the desired information is available. ^−^The information is not available. ^?^No statement about disclosure/conflict of interest present. ^a^Disclosure statement/conflict of interest is positive but does not affect, in our understanding, the results.

In the following, we will sort the publications by the main source of EVs investigated in the studies and extracted the EV purification method (detailed description in supplemental part), size, injury model, if applicable the main effector in EVs as well as the investigated EV marker.

### Results per EV source

#### Cardiomyocytes

Cardiomyocytes are, next to fibroblasts and endothelial cells, one of the most abundant cell types in the mammalian heart. Due to their importance in cardiac function, researchers are extensively studying their physiological properties^52^ as well as their capabilities to secrete EVs^33^. Publications investigating cardiomyocyte derived EVs are summarized in Table 2.

**Table 2:** Publications investigating EVs from cardiomyocytes.

Ref.	PM	Size	Injury model	Main effector	EV marker
Garcia *et al*.^21^	UC, sucrose cushion, precipitation	≈50–100 nm*	NSI	NSI	CD63, CD9, CD81
Borosch *et al*.^33^	Size-exclusion chromatography, UC	≈150 nm	NSI	NSI	Alix, HSP70, CD63, Flot-1, CD81
Ribeiro-Rodrigues *et al*.^30^	UC	111–137 nm	MI, H_2_O_2_	miR-222, miR-143	Alix, HSP70, CD63, Flot-1, TSG-101, CD81, GAPDH
Zhang *et al*.^43^	Precipitation	NSI	NSI	(HSP20)	NSI
X. Wang *et al*.^24^	UC	54.9–55.2 nm	Streptozotocin induced diabetes	HSP20	CD63, CD81, HSP70

EV-purification method (PM), particle size, investigated injury model, main effector mediating protection are stated and EV marker. UC = ultracentrifugation; HSP = heat shock protein; MI = myocardial infarction; NSI = not specifically indicated; Ref. = reference; TSG = tumour susceptibility gene; Flot = flotillin; GAPDH: glyceraldehyde 3-phosphate dehydrogenase; *self-assessed (assumption made from EM images).

Garcia *et al*. showed that starvation of the immortalized cardiomyocyte cell line H9c2 increased the secretion of EVs with altered composition and enhanced capability to induce tube formation^21^. Borosch *et al*. investigated the EV composition of primary cardiomyocytes and H9c2 cells after preconditioning with hypoxia or isoflurane which resulted in significantly altered cargo composition of the cell-derived EVs^33^. A similar study confirmed that EVs from ischemic cardiomyocytes protected against oxidative-induced lesion, promoted angiogenesis and proliferation of endothelial cells *in vitro*. The authors suggested that miR-222 and miR-143, encapsulated in hypoxic EVs, are partially responsible for the pro-angiogenic effects. *In vivo* experiments confirmed enhanced angiogenesis due to hypoxic EV treatment after MI but no reduction of fibrosis was observed^30^.

Zhang *et al*. identified HSP20, as a possible mediator of cardioprotection transferred by EVs^43^. The authors postulated that HSP20-overexpressing primary cardiomyocytes secrete EVs with elevated levels of HSP20 compared to EVs from control cells. HSP20 additionally promoted proliferation, migration and tube formation. Unfortunately, due to methodological limitations in this study, not all observed effects can be attributed to HSP20 in EVs. Basic EV-related experiments such as EM-images and testing for EV markers were not conducted in this study^43^.

In a following publication, the authors investigated whether cellular HSP20 overexpression and thereby elevated HSP20 levels in EVs might protect the myocardium in diabetes^24^. Compared to EVs from control cells, EVs secreted from cardiomyocytes^HSP20^ exhibited elevated levels of p-protein kinase B (pAkt), survivin and superoxiddismutase 1 (SOD1) and protected against *in vitro* hyperglycemia-triggered cell death^24^.

#### Cardiac progenitor cells

Cardiac progenitor cells (CPCs) represent a heterogeneous group of cells throughout the heart and the surrounding vessels which can be activated upon injury and contribute to the cardiac renewal^53,54^. Recent findings indicated that CPC-derived EVs might have a predominant role in transmitting cardioprotective mediators to the damaged heart. With our defined search criteria, we found four articles investigating the protectivity of CPC-derived EVs (Table 3).

**Table 3:** Publications investigating EVs from cardiac progenitor cells (CPCs).

Ref.	PM	Size	Injury model	Main effector	EV marker
Barile *et al*.^13^	Precipitation, UC	30–90 nm	Starvation, MI	miR-210, miR-132,	CD63, CD9, CD81
Chen *et al*.^34^	Precipitation	40–100 nm	H_2_O_2_, I/R-injury	(miR-451)	CD63
Xiao *et al*.^39^	Precipitation	50–150 nm	H_2_O_2_	miR-21	Alix, CD63, CD9
Gray *et al*.^22^	UC	96–102 nm	I/R injury	(11 miRNAs)	CD9

EV-purification method (PM), particle size, investigated injury model, main effector mediating protection and EV marker are stated. I/R = ischemia/reperfusion; UC = ultracentrifugation; MI = myocardial infarction; NSI = not specifically indicated; Ref. = reference.

Barile *et al*. isolated CPCs from patients who underwent heart valve surgery^13^. Apoptosis was reduced in the starved and reperfused immortalized cardiomyocyte HL-1 cell line, which were treated with CPC-derived EVs. Additionally, tube formation in in human umbilical vein endothelial cells (HUVECs) and angiogenesis *in vivo* were enhanced by those EVs. *In vivo* experiments indicated that a treatment with EVs improved the left ventricular ejection fraction (LVEF) and reduced scar tissue after MI. Levels of miR-210 and miR-132, were elevated in CPC-derived EVs compared to EVs from fibroblasts. The authors suggested that these miRNAs down-regulate ephrin A3, protein-tyrosine phosphatase 1 (PTP1) and RasGTPase-activating protein (RasGap)-p120 and thereby transduced their beneficial effects in the recipient cells and tissue^13^.

In a similar study, the authors challenged H9c2 cells with H_2_O_2_ and performed an *in vivo* model of I/R injury^34^. CPC-derived EVs were again able to attenuate apoptosis, reduced the amount of terminal deoxynucleotidyl transferase dUTP nick end labelling (TUNEL) positive cells and pro-apoptotic caspase 3/7 activation. Additionally, the transcription factor GATA4-responsive miR-451 was overexpressed in CPC-derived EVs^34^.

Xiao *et al*. used an oxidative stress model to assess the protective properties of CPC-derived EVs^39^. First, the authors observed that H_2_O_2_ stressed CPCs secreted more EVs and the cargo of those EVs was also altered compared to EVs derived from untreated cells. EVs from treated cells had increased levels of miR-21, targeting programmed cell death protein 4 (PDCD4), which is involved in apoptosis. The authors could show that H9c2 cells, which were pre-treated with CPC-derived EVs, were more resistant to H_2_O_2_ treatment. Interestingly, EVs from pre-treated source were even more protective, presumably due to the elevated miR-21 levels and thereby reduced PDCD4 levels in the recipient cells^39^.

Gray *et al*. investigated EVs derived from hypoxia stimulated CPCs, that were able to promote tube formation and decreased profibrotic gene expression^22^. Hypoxic treatment of the cells indeed altered the EV composition. 11 miRNAs were upregulated in EVs derived from hypoxic CPCs compared to EVs, which were isolated from normoxic cells. Additionally, those EVs were able to improve cardiac function in a model of I/R-injury. EVs from CPCs, which were previously subjected to 12 h hypoxia, were able to reduce fibrosis *in vivo*. EVs from cells, which were not treated with hypoxia or experienced a shorter treatment, had attenuated effects^22^.

#### Cardiosphere derived cells

Cardiac surgical biopsy specimens exhibit the potential of secreting a heterogeneous population of cardiac cells called cardiosphere derived cells (CDCs)^55^. Publications matching our search criteria and investigating EVs from these cells are stated in Table 4.

**Table 4:** Publications investigating EVs derived from cardiohere derived cells.

Ref.	PM	Size	Injury model	Main effector	EV marker
Ibrahim *et al*.^35^	Precipitation	30–90 nm	MI	miR-146a	CD63
Namazi, Namazi *et al*.^25^	UC	150–170 nm	cobalt chloride as hypoxia mimetic agent	NSI	CD63, CD81
Namazi, Mohit *et al*.^28^	UC	140–180 nm	NSI	NSI	CD63, CD81
de Couto *et al*.^47^	Precipitation	150 nm	I/R injury	miR-181b	CD63, Alix, Hsp70

EV-purification method (PM), particle size, investigated injury model, main effector mediating protection and EV marker are stated. I/R = ischemia/reperfusion; UC = ultracentrifugation; MI = myocardial infarction; NSI = not specifically indicated; Ref. = reference; HSP = heat shock protein.

EVs from CDCs inhibited apoptosis and promoted proliferation in neonatal cardiomyocytes^35^. *In vivo* data also demonstrated reduced scar mass, resulting in elevated contractility and increased viable mass in a MI-injury model upon treatment with CDC-derived EVs. Blocking the generation of EVs with GW4869 *in vitro* and *in vivo* resulted in enhanced apoptosis, diminished cardiomyocytes proliferation, increased scar mass and reduced function of the heart. The authors identified miR-146a as key mediator of cardioprotection^35^. GW4869 is able to inhibit sphingomyelinases, thereby blocking the ceramide-dependent budding of intraluminal vesicles into the lumen of MVBs which reduces biogenesis of EVs^56,57^. CDC-derived EVs additionally decreased caspase 3/7 activity in human embryonic stem cell-derived cardiomyocytes upon cobalt chloride treatment^25^ and enhanced tube formation in HUVECs^28^. The *in vivo* relevance of CDC derived EVs was confirmed by a study conducted in 2017 showing that EV associated miR-181b could decrease the infarct size after I/R injury^47^.

#### Fibroblasts

The main function of fibroblasts is to produce extracellular matrix and thereby stabilize the surrounding tissue^58^. New studies attribute those cells to far more complex signalling within the heart which is in part based on EVs. Recent findings indicated that fibroblast-derived EVs might contribute to cell migration and proliferation of cardiac fibroblasts whereas others demonstrated a detrimental impact^17,33^. Publications matching our search criteria and investigated fibroblast-derived EVs are summarized in Table 5.

**Table 5:** Publications investigating fibroblast-derived EVs.

Ref.	PM	Size	Injury model	Main effector	EV marker
Bang *et al*.^17^	UC	50–100 nm	Left ventricular pressure overload	miR-21-3p	CD63, GAPDH
Ibrahim *et al*.^35^	Precipitation	NSI	MI	NSI	CD63
Y. Wang *et al*.^45^	Precipitation	100 nm	H_2_O_2_, I/R-injury	NSI	CD63, TSG101
Barile *et al*.^13^	Precipitation, UC	NSI	Starvation, MI	NSI	NSI
de Couto *et al*.^47^	Precipitation	150 nm*	I/R Injury	NSI	NSI
Borosch *et al*.^33^	Size-exclusion chromatography	≈150 nm	NSI	NSI	Alix, HSP70, CD63, Flot-1, CD81

EV-purification method (PM), particle size, investigated injury model, main effector mediating protection and EV marker are stated. UC; ultracentrifugation, MI; myocardial infarction. I/R = ischemia/reperfusion; UC = ultracentrifugation; NSI = not specifically indicated; Ref. = reference; TSG = tumour susceptibility gene; Flot = flotillin; GAPDH: glyceraldehyde 3-phosphate dehydrogenase; *Self-assessed (Assumption from Supplemental Information).

Cardiac fibroblast-derived EVs induced pathological hypertrophy in cardiomyocytes *in vitro*^17^. miR-21-3p was identified as specific mediator in those EVs which targets sorbin and SH3 domain-containing protein 2 (SORBS2) and PDZ and LIM domain 5 (PDLIM5) inducing hypertrophy^17^. Others showed that EVs from fibroblasts had diminished protective capabilities. Ibrahim *et al*. demonstrated the benefits of EVs from cardiosphere derived cells (CDCs) by different *in vivo* and *in vitro* experiments. In contrast, normal human dermal fibroblasts (NHDFs) were not able to transmit comparable protection^35^. These findings were supported by others^47^. Wang *et al*.^45^ additionally supported this notion by demonstrating that EVs from induced pluripotent stem cells (iPS) were able to protect against myocardial I/R-injury while EVs from cardiac fibroblast had a diminished effect. Nevertheless, cardiac fibroblast-derived EVs significantly reduced caspase 3/7 activity after H_2_O_2_ treatment in H9c2 cells compared to control^45^ and enhanced proliferation/migration in cardiac fibroblasts^33^.

#### Mesenchymal stem cells

Mesenchymal stem cells (MSCs) are adult stem cells with the potential to differentiate into multiple other cell types. The tremendous capabilities of MSCs are also attributed to their great potential of secreting important factors for the control of haematopoiesis or immunomodulation^59^. We found several publications fitting our search criteria and investigated whether MSC-derived EVs promote protection (Table 6).

**Table 6:** Publications investigating EVs from mesenchymal stem cells.

Ref.	PM	Size	Injury model	Main effector	EV marker
Feng *et al*.^10^	UC/Precipitation	30–120 nm	MI	miR-22	CD63
X. Wang *et al*.^9^	UC, sucrose gradient	10–100 nm	Sepsis/Inflammation	miR-223	CD81, CD63
Teng *et al*.^44^	Precipitation	50–100 nm	MI	NSI	CD63
Zhang *et al*.^46^	Precipitation	11–98 nm	MI	NS	CD63, GAPDH
Zhao *et al*.^23^	UC, sucrose cushion, ultrafiltration	20–85 nm	*In vitro* hypoxia, MI	NSI	CD9, CD63
Kang *et al*.^36^	Precipitation	40–90 nm	MI	CXCR4	CD9, CD63
Yu *et al*.^41^	Precipitation	≈100 m*	NSI	(miR-221)	CD9, CD63, HSP70
Yu *et al*.^42^	Precipitation	≈100 nm	*In vitro* hypoxia, MI	miR-19a	CD9, CD63, HSP70
Lai *et al*.^20^	Size exclusion fractionation (UC sucrose gradient, Immunoprecipitation)	55–65 nm	I/R-injury	NSI	CD9, CD81, Alix
Arslan *et al*.^50^	Ultrafiltration, Chromatography	NSI	I/R-injury	NSI	NSI
Shi *et al*.^48^	Precipitation	30–100 nm	H_2_O_2_	miR-21	CD9, CD63, HSP70

EV-purification method (PM), particle size, investigated injury model, main effector mediating protection and EV marker are stated. CXCR = CXC chemokine receptor; UC = ultracentrifugation; MI = myocardial infarction; I/R = ischemia/reperfusion; NSI = not specifically indicated; Ref. = reference; HSP = heat shock protein; GAPDH: glyceraldehyde 3-phosphate dehydrogenase *self-assessed (assumption made from EM images).

Feng and Co-workers identified miR-22 as potential cardioprotectant which was secreted via EVs from ischemic-preconditioned MSCs^10^. EVs from these cells were able to reduce fibrosis in infarcted hearts. Methyl CpG binding protein (Mecp2) is a direct target of miR-22, which was enriched in the investigated EVs and contributed to reduced cardiac damage^10^.

MSC-derived EVs also contributed to cardioprotection in a sepsis model induced by cecal ligation and puncture. EV treatment increased the ejection fraction of mice and improved the survival in polymicrobial sepsis^9^. The authors could demonstrate that tumor necrosis factor-α (TNFα), interleukin 6 (IL-6) and IL-1β secretion was reduced in macrophages after the treatment with MSC-derived EVs *in vitro*. The authors attributed the cardioprotective properties to miR-223 from WT-MSC derived EVs and thereby targeting Semaphorin-3A (Sema3A) and Signal transducer and activator of transcription 3 (Stat3)^9^. In addition to enhanced HUVEC tube formation and reduced fibrosis, Teng and Co-workers could show that MSC-derived EVs reduced the inflammation in the infarcted area^44^. These results were supported by an *in vivo* model of MI where MSC-derived EVs increased cardiac stem cell tube formation and reduced fibrosis. The authors suggested that this distinct miRNA cargo might be the reason for the cardioprotective properties of MSC-derived EVs^46^. Additionally, in a model of MI, administration of EVs reduced cardiac damage and improved systolic function^23^.

In comparison to EVs from wild types, transfection enforced expression of specific proteins in host cells and might further enhance the resulting EV capabilities. EVs derived from MSC^CXCR4^ were able to reduce caspase 3 activity and induced upregulation of IGF-1α and pAkt in neonatal cardiomyocytes^36^. Implantation of a cell patch, which was treated with EVs from MSC^CXCR4^, was significantly more potent to reduce the infarct size compared to cell patches with control EVs. These data provided evidence that the protectivity from MSC-derived EVs may be enhanced by specific cargo loading^36^. In addition, miR-221 was investigated as EV-mediated cardioprotective factor. Rat ventricle cardiomyocytes which were cultivated under hypoxic conditions were more robust against this stimulus when incubated with supernatant from MSC^GATA-4^ ^41^. However, the experiments conducted in this study do not allow the conclusion that MSC-derived EVs are protective. For instance, isolated EVs were not transferred to other cells to investigate their ability of cytoprotection^41^. In a study conducted two years later, EVs from MSC^GATA-4^ protected neonatal cardiomyocytes from hypoxia-induced cell death^42^. The cardiac function, after ligation of the left anterior descending coronary artery, was also improved. miR-19a, which was enriched in EVs from MSC^GATA-4^, was identified as the effector mediating the protection^42^. miR-19a targets phosphatase and tensin homolog (PTEN), inhibiting cell proliferation and induces apoptosis^60^. Even though the authors showed that EVs from MSC^GATA-4^ mediated improved cell function and protection, EVs from control MSCs were still protective as well^42^. In an early study conducted in 2009, Lai and co-workers investigated the protective effects of EVs secreted from human embryonic stem cell-derived mesenchymal stem cells (HuES9.E1). Isolated EVs were able to reduce the infarct size after myocardial I/R injury^20^. The underlying signalling pathways include decreased oxidative stress as well as increased Akt and glucogen synthase kinase-3α/β phosphorylation (GSK-3α/β)^50^. EVs from, previously with H_2_O_2_ treated, MSCs additionally contributed to reduced oxidative stress induced cell death by inhibition of PTEN. miR-21 was identified as key mediator of those protective properties^48^.

#### Body fluids

In contrast to the previously investigated EV sources, the original sources of EVs in body fluids are diverse. Numerous publications were identified by our search criteria, studying EVs from different body fluids and are further investigated in the following (Table 7).

**Table 7:** Publications investigating EVs derived from several body fluids.

Ref.	Source	PM	Size	Injury model	Main effector	EV Marker
Vicencio *et al*.^7^	Plasma	UC	75 ± 2 nm rat plasma 75 ± 7 nm human plasma	I/R injury	HSP 70	CD63, CD81, HSP70
Li *et al*.^37^	Serum	Precipitation	50–400 nm	I/R injury	(miR-144)	CD63
Ma *et al*.^15^	Plasma	UC	367.6 nm	I/R Injury	NSI	CD41, Annexin V
Minghua *et al*.^49^	Plasma	Precipitation	50–200 nm	H_2_O_2_ treatment, I/R injury	miR-24	CD63, CD81, CD9
Giricz *et al*.^8^	Coronary perfusates	UC	10–1000 nm	I/R injury	NSI	HSP60
Svennerholm *et al*.^16^	Plasma	UC, sucrose gradient	30–350 nm	NSI	NSI	CD81
Yamaguchi *et al*.^40^	Serum	UC	NSI	MI	(miR-29a)	CD9, HSP90, GAPDH
X. Wang *et al*.^24^	Serum	Precipitation	NSI	Streptozotocin induced diabetes	HSP20	NSI
Davidson *et al*.^27^	Plasma	UC	≈100 nm	Hypoxia reoxygenation	NSI	CD81, HSP70
Wider *et al*.^32^	Serum	UC	0–200 nm	Hypoxia reoxygenation	NSI	Flot-1, HSP60
Svennerholm *et al*.^31^	Plasma	UC, sucrose gradient	30–350	NSI	NSI	CD81
Cheow *et al*.^18^	Plasma	UC	50–100 nm	NSI	NSI	CD9, CD81
Obata *et al*.^29^	Plasma	Precipitation, UC	NSI	NSI	NSI	Syntenin

EV-purification method (PM), particle size, investigated injury model, main effector mediating protection and EV marker are stated. UC = ultracentrifugation; MI = myocardial infarction; I/R = ischemia/reperfusion; HSP = heat shock protein; NSI = not specifically indicated; Ref. = reference; Flot = flotillin; GAPDH: glyceraldehyde 3-phosphate dehydrogenase.

Vicencio *et al*. hypothesized that an established cardioprotective treatment has an impact on EVs and their cargo^7^. The authors analysed whether blood derived EVs from a remote ischemic preconditioned (rIPC) donor were more protective than those from an untreated source. Surprisingly, several *in vitro* and *in vivo* experiments revealed that EVs from treated and untreated source were protective in a similar fashion. EVs in general were able to reduce cell death and ultimately the infarct size. The authors suggested that HSP70 on the EV surface, might interacted with toll-like receptor 4 (TLR-4) on the recipient cells, thereby triggering a signal cascade which activates intracellular HSP27 which further promotes cardioprotection^7^. In a similar study, EVs from a rIPC group and the corresponding control group were isolated from serum and analysed^37^. The predicted effector, miR-144 was not upregulated upon rIPC treatment in EVs but in the serum of the treated animals. In contrast, the precursor form of miR-144 was enriched in EVs. The authors suggested that miR-144 is important for cardioprotection but EVs are probably not the main carrier and mediator of this protective miRNA^37^. A similar study revealed that EVs, isolated from rIPC-rats, could reduce the infarct size in a model of *in vivo* I/R injury^15^. Minghua *et al*. supported these findings by demonstrating that EVs from rIPC rats decreased apoptosis in an *in vitro* H_2_O_2_ stress model as well as decreased infarct size in an I/R injury *in vivo* model. The authors suggested that miR-24 encapsulated in EVs is able to transduce the protective properties^49^. A different approach investigated EV mediated protection in an *ex vivo* IPC Langendorff model. The perfusates from preconditioned rat hearts were collected and used to treat hearts prior to infarction. EV-depleted perfusates caused increased infarct size compared to the EV-containing perfusates^8^. In another IPC study, the authors investigated whether this treatment might promote a change in the DNA content in EVs. The authors could not detect any difference in the number of sequenced gene fragments between treatment and control^16^. In a rat model, rIPC resulted in an increase of miR-29a in serum-derived EVs^40^. miR-29a is a key regulator of tissue fibrosis and the increase of this miRNA might contribute to the finding of reduced fibrosis after rIPC treatment. Nevertheless, the exclusive protectivity of EVs was not investigated^40^. As mentioned previously, HSPs, in or on the surface of EVs, might mediate cardioprotection. Wang *et al*. developed a transgenic mouse model with cardiac specific overexpression of HSP20^24^. EVs isolated from mouse^HSP20^ serum had higher HSP20 levels compared to EVs from control mice. The cardiac contractile function of diabetic mice^HSP20^ was also improved compared to control mice. Attenuating the release of EVs by GW4869 *in vivo*^61^ resulted in reduced HSP20-mediated cardiac function, evaluated by left ventricular internal dimension-diastole (LVIDd) and LVEF in diabetic mice^24^.

EVs from diabetic rats or patients were not able to protect cardiomyocytes from hypoxia/reoxygenation injury *in vitro*. EVs from healthy donors instead did^27^. Similar results were obtained in a study subjecting healthy and diabetic rats to rIPC and evaluating the protectivity in an *in vitro* model of hypoxia reoxygenation. Cell death of HL-1 cells was reduced if treated with EVs from healthy rats but no effects were observed with EVs from diabetic rats^32^.

An observative study was conducted in 2016, comparing the plasma EV cargo of patients with MI and patients with stable angina. Indeed, the authors identified several EV proteins which were upregulated upon MI^18^. A similar study investigated in a porcine *in vivo* model the influence of ischemic preconditioning on EV cargo. EVs from preconditioned animals had an altered mRNA cargo related to proteins which are commonly associated with the protective effects of ischemic preconditioning^31^.

#### Other cell types

Several studies which were found by our search criteria did not fit in the previously described groups. We will therefore describe the benefits of EVs from these sources in Table 8.

**Table 8:** Publications investigating EVs from cells not fitting in the previously described categories.

Ref.	Source	PM	Size	Injury model	Main effector	EV marker
Y. Wang *et al*.^45^	Induced pluripotent stem cells	Precipitation	100 nm	I/R-injury	(miR-21, miR-210)	CD63, TSG101
T. Kang *et al*.^14^	Adipose-derived stem cells	UC	<1 µm	NSI	miR-31	Alix
Gu *et al*.^19^	Endothelial progenitor cells	UC	NSI	Angiotensin II-Induced hypertrophy	NSI	NSI
Vandergriff *et al*.^51^	Cardiac stem cells	Ultrafiltration	129.6 nm	doxorubicin induced cardiomyopathy	NSI	CD63
Ong *et al*.^38^	Endothelial cells	Precipitation	30–110 nm	Hypoxic stress *in vitro*	miR-126, miR-210	CD63, CD9
Balbi *et al*.^26^	Human amniotic fluid stem cells	UC	50–200 nm	Muscle atrophy, H_2_O_2_	NSI	TSG101, Alix, CD81, CD9, CD63, Annexin V
Davidson *et al*.^27^	HUVECs	UC	≈100 nm	Hypoxia reoxygenation	NSI	CD81
Obata *et al*.^29^	Endothelial F2 cells	UC, density gradient,	≈90 nm*	NSI	NSI	Alix, HSP70, CD63 syntenin

EV-purification method (PM), particle size, investigated injury model, main effector mediating protection and EV marker are stated. UC = ultracentrifugation; I/R = ischemia/reperfusion; NSI = not specifically indicated; Ref. = reference; TSG = tumour susceptibility gene; HSP = heat shock protein; *self-assessed (assumption made from nanoparticle tracking analysis).

IPS cells transduce their beneficial properties also through EVs. *In vitro* experiments indicated that iPS-derived EVs inhibit proapoptotic caspase 3/7 activation after H_2_O_2_ treatment of H9c2 cells^45^. The conducted experiments also identified two specific miRNAs miR-21 and miR-210 which potentially transmitted the cardioprotective properties of iPS cell-derived EVs although no confirmation experiments were performed. In an *in vivo* model of I/R injury, apoptosis of cardiomyocytes was additionally reduced after treatment with iPS-derived EVs^45^.

Recently adipose tissue has proven to be a reliable source of stem cells^62^. Kang *et al*. were able to show that EVs from adipose-derived stem cells (ASCs), preconditioned with endothelial differentiation medium, induced HUVEC tube formation. miR-31 was identified as mediator of these pro-angiogenic effects by targeting the factor-inhibiting hypoxia inducible factor-1 (HIF-1) (FIH1)^14^.

Gu and co-workers performed several *in vitro* experiments to investigate whether EVs from endothelial progenitor cells (EPCs) might protect H9c2 cells from angiotensin II induced hypertrophy^19^. Apoptosis and cell viability were improved by EPC-derived EVs. Additionally, the isolated EVs induced phosphorylation of Akt and endothelial nitric oxide synthase (eNOS) in angiotensin II treated H9c2 cells^19^.

The beneficial effects of EVs from cardiac stem cells were investigated in a mouse model of doxorubicin induced dilated cardiomyopathy. Mice received cardiac stem cell-derived EVs which were able to improve cardiac function, reduce fibrosis in the myocardium as well as TUNEL positive cells, respectively DNA fragmentation^51^.

Endothelial cells, overexpressing HIF-1 secreted EVs with higher contents of miR-126 and miR-210. The specific cargo of these EVs resulted in an activation of pro-survival kinases and induced a glycolytic switch in the recipient CPCs. EVs additionally reduced the cellular damage during hypoxic conditions *in vitro*^38^. Human amniotic fluid stem cells (hAFS) secreted EVs which were able to mediate antiapoptotic effects *in vitro*. Hypoxic preconditioning of hAFS additionally enhanced the protectivity of EVs and furthermore modulated the miRNA cargo of those EVs^26^. Surprisingly, EVs from HUVECs, cultivated under hyperglycaemic conditions, were not able to protect primary adult cardiomyocytes from hypoxia-reoxygenation whereas EVs from regular cultivated cells were protective^27^. In a recent study, Obata demonstrated that adiponectin is able to stimulate ceramide secretion by EVs, reducing the intracellular level of ceramides *in vitro* and *in vivo*^29^.

### Summary of EV related benefits and meta-analysis

**Figure 2:**
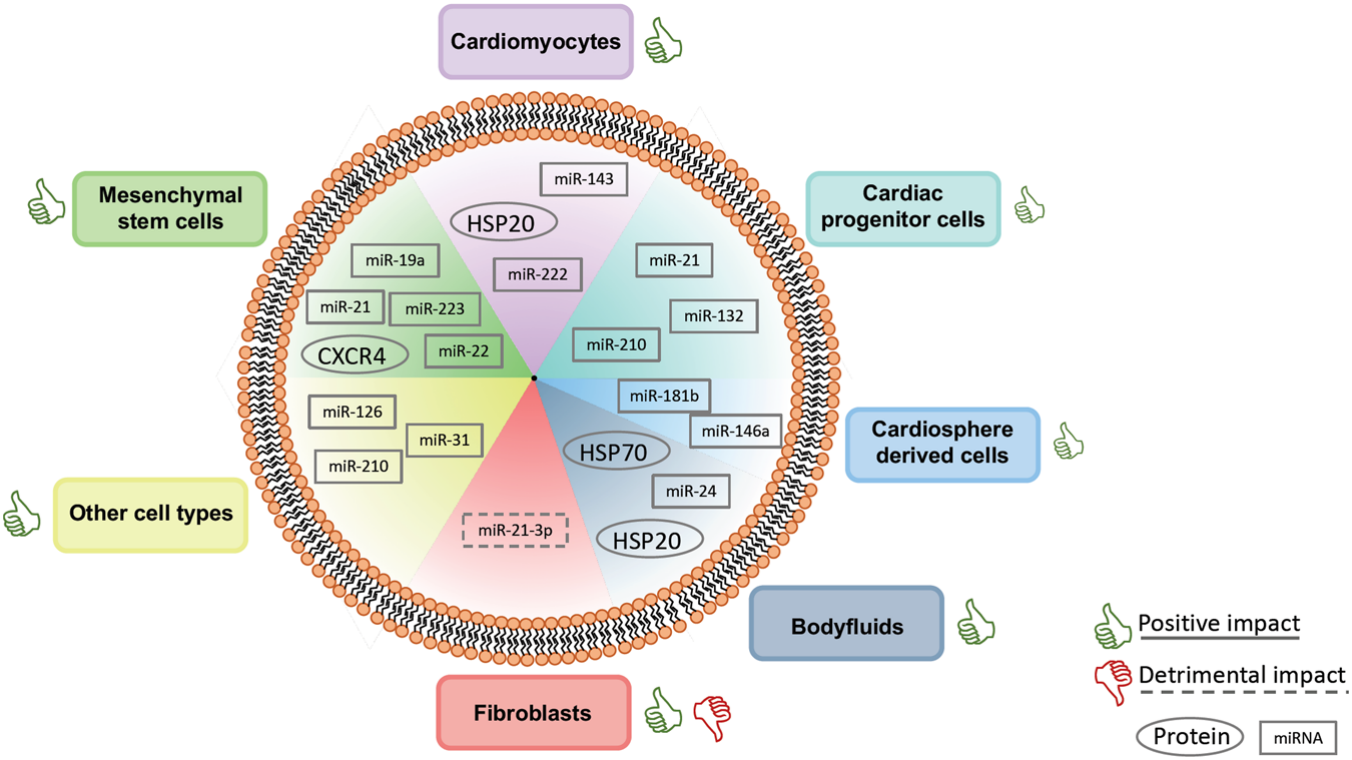
Summary of in EVs encapsulated molecules mediating cardio protective or detrimental impact. Molecules are ordered by origin. Thumbs up indicate a general positive impact of EVs derived from the particular source. Thumps down indicate a detrimental impact. Effector molecules surrounded by solid lines are specific for a positive impact whereas a dashed line stands for molecules with a negative impact. Proteins are surrounded by a circle, miRNAs by a rectangle.

We additionally summarized the type of performed experiments to investigate the protective properties of EVs and recapitulated the beneficial outcomes in Table 9. Almost all EVs, from all sources, were able to mediate protection. Several publications investigated, whether overexpression of specific molecules results in EVs with enhanced protective properties e.g. enhanced angiogenesis or reduction of apoptosis^17,24,36,38,42^. Others investigated the capabilities and characteristics of EVs secreted under regular conditions^7–10,13–36,39–42,44–51^. Different treatments of the EVs source also enhanced EV properties^8,10,14–16,21,22,28–33,39,40,48,49^. Researchers investigating fibroblast-derived EVs postulated that they might not contribute to cardioprotection in the same extent^13,17,35,45,47^ whereas other groups observed enhanced proliferation/migration after treatment with fibroblast-derived EVs^33^. We summarized the major effector molecules mediating protective or detrimental properties in Fig. 2.

**Table 9:** Summary of all publications which investigated the protective effects of extracellular vesicles (EVs) sorted by the EV source.

EV source	Publication	Cardiac damage	Apoptosis/cell death	Caspase activation	Proinflammatory mediators	LDH release/Activity	Ejection fraction	Protective kinases	Proliferation/Migration	Tube formation/cell survival
CPCs	Barile *et al*.^13^	X	X	X			X			X
	Chen *et al*.^34^	X	X	X						
	Xiao *et al*.^39^		X	X						
	Gray *et al*.^22^	X								X
CDCs	Namazi, Namazi *et al*.^25^		X	X						
	Ibrahim *et al*.^35^	X	X		X		X		X	X
	Namazi, Mohit *et al*.^28^									X
	de Couto *et al*.^47^	X	X		X		X		X	
MSCs	Feng *et al*.^10^	X	X							
	X. Wang *et al*.^9^	X	X		X		X			X
	Kang *et al*.^36^	X	X	X			X	X		X
	Teng *et al*.^44^	X			X		X			X
	Zhang *et al*.^46^	X					X		X	X
	Zhao *et al*.^23^	X	X				X		X	X
	Yu *et al*.^42^	X	X			X	X	X		X
	Arslan *et al*.^50^	X			X		X	X		
	Lai *et al*.^20^	X								
	Shi *et al*.^48^		X	X				X		
Fibroblasts	Borosch *et al*.^33^								X	
Cardiomyocytes	X. Wang *et al*.^24^	X	X				X	X	X	X
	Garcia *et al*.^21^								X	X
	Ribeiro-Rodrigues *et al*.^30^								X	X
Body fluids	Vicencio *et al*.^7^	X	X					X		
	Ma *et al*.^15^	X								
	X. Wang *et al*.^24^	X	X				X			
	Giricz *et al*.^8^	X								
	Davidson *et al*.^27^		X					X		
	Minghua *et al*.^49^	X	X	X		X	X			
	Wider *et al*.^32^		X							
Other cell types	Y. Wang *et al*.^45^	X	X	X						
	Balbi *et al*.^26^		X		X				X	X
	Kang *et al*.^14^								X	X
	Gu *et al*.^19^		X					X		X
	Vandergriff *et al*.^51^	X	X				X			
	Ong *et al*.^38^					X		X		X
	Davidson *et al*.^27^		X							

The effect of EVs derived from the indicated source are indicated as follows: ^↓^Indicates a reduction, ^↑^indicates an elevation/activation. LDH: Lactate dehydrogenase, CPCs: cardiac progenitor cells, CDCs: cardiosphere derived cells, MSCs: mesenchymal stem cells.

**Figure 3:**
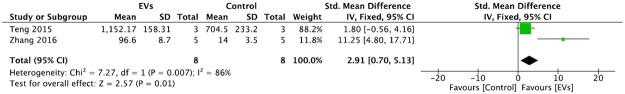
Forest plots showing the results of meta-analysis of the effect of formation of new capillaries upon a treatment with EVs. Data are expressed as standard mean difference with 95% confidence interval.

**Figure 4:**
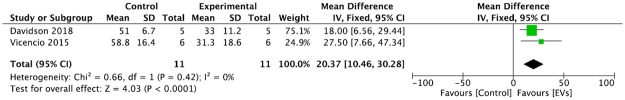
Forest plots showing the results of meta-analysis of the effect of protecting cardiomyocytes from hypoxia-reoxygenation injury after a treatment with EVs. Data are expressed as mean difference with 95% confidence interval.

To evaluate whether the described properties of EVs are indeed cardioprotective we performed a meta-analysis. Pooling two independent studies^44,46^ which investigated the number of capillaries after EV treatment (Fig. 3) and two studies investigating the protective effect of EVs in a setting of hypoxia-reoxygenation (Fig. 4)^7,27^. Both analysis indicated significant effects favouring EV treatment.

## Discussion

This is the first all-encompassing systematic review/meta-analysis investigating the cardioprotective effects of EVs. 43 studies were chosen for analysis and data extraction. We found that EVs derived from different cell types as well as from different body fluids, mediated beneficial properties. Only EVs from fibroblasts had, as described in some investigated studies, harmful effects and mediated hypertrophy. We evaluated the EV specific experiments, investigating the predominant mediators of protection carried by EVs and categorized the studies by EV source and the experiments performed to investigate the positive capabilities of EVs. We finally conducted a meta-analysis and verified the positive properties of EVs by combining the results of independent studies.

Investigating EVs and their beneficial properties is often challenging and several basic experiments are needed to ensure that the described effects in the corresponding investigation are transferred by EVs. Recommendations, first described in 2013, what kind of experiments are needed or which isolation or purifications methods are suitable to ensure an appropriate EV preparation were given by several publications^11,12,63–65^. Nevertheless, some of these guidelines have been published only recently and several of the here included publications were published before these guidelines. These guidelines are constantly changing due to novel developments and new insights in the field of EV research, making it impossible to introduce guidelines for a broader group of researchers. Applying those, partly strict, criteria to all investigated publications in our review may therefore not be suitable. Nevertheless, EM images and identification of EV-marker are in our understanding mandatory in EV research.

EVs from numerous sources were successfully isolated by the included studies. Few studies did not perform the necessary experiments to ensure an appropriate EV preparation as described previously^11,63,64^. We have to mention that the existing methods to verify EV properties are far from absolute. For instance, techniques to measure the concentration of EVs or to describe the morphological properties might not distinguish between EVs and particles with a similar size range, as reviewed by others^66^. The indicated sizes, stated in the results part, have also to be considered as a range since EVs with only one size cannot be isolated so far. Only a variety of different experiments is suitable to absolutely ensure that the isolated particles are indeed EVs. Additionally, precipitation and basic ultracentrifugation methods might result in co-isolation of non-EV particles and thereby hold the risk for impurities^67,68^. A study considering these problems was performed in 2016, investigating EV marker after different centrifugation steps in the resulting pellets^64^. Due to the great variety and no gold standard EV purification protocol, we therefore only distinguished between UC, precipitation-based and other protocols in our systematic review. Further research and development of more appropriate methods for EV purification and detection of multiple EV sources are needed to ensure comparable results from different studies. We would like to point out that in some of the described studies immortalized cell lines such as H9c2 and HL-1 cells were used to investigate EV properties. For instance, undifferentiated H9c2 cells might not represent a cardiac specific phenotype and the results might therefore be met with caution^69,70^.

The cardioprotective properties of EVs from different sources have been investigated by several studies included in this review. The main effector of these benefits are miRNAs such as miR-210 or miR-132, inhibiting apoptosis or enhancing tube formation^13^. Especially miR-21 and miR-210 are encapsulated in EVs from numerous sources and mediated protection in different ways. The beneficial effects of EV-derived miRNAs in other diseases such as autoimmune hepatitis^71^ or sepsis^72^ has additionally shown by other groups. The conclusions whether EVs from genetically engineered cells, from a treated donor or regularly secreted are cardioprotective, are inconsistent. In several studies which investigated EVs from a genetically engineered origin, the EVs from control cells had also beneficial capabilities, even if the effects were not that distinct. These observations were also made in studies investigating the EVs from previously treated sources such as ischemic preconditioning. We therefore conclude that EVs in general are protective and that these properties might be enhanced by an appropriate treatment of the EV source or transfection of the host cells. EVs from several body fluids have also been proven to mediate positive properties. These EVs might originate from different sources making it difficult to identify specific molecules mediating the protective effects. The results have therefore to be met with caution and further *in vitro* analysis might be needed to investigate which treatment triggers the release of EVs from a distinct cell type mediating the protection.

Even though the same cell types were investigated in different studies and similar experiments were performed to evaluate the EV mediated protection, a stringent meta-analysis was not possible due to the lack of consistency. Tube formation or cell survival experiments were conducted by numerous studies. But these, for example, were performed with MSC-derived EVs either from genetically engineered or wild type cells or with different tube forming cells^36,44,46^. To evaluate exemplarily the protective properties of EVs, we combined the data of two studies investigating the formation of capillaries in the heart after EV treatment. One publication evaluated the number of capillaries after direct EV treatment whereas the second article investigated if cells, previously treated with EVs, promote angiogenesis after implantation into the heart^44,46^. Combination of both data sets revealed significant difference favouring EV treatment. The meta-analysis from two different studies conducted by the same group indicated that EVs protect cardiomyocytes from hypoxia/reoxygenation injury^7,27^.

Taken together, these findings demonstrate the urgent need for more consistency and adequately designed studies, not only for the EV-purification methods but also for the performed experiments investigating the effect of EVs.

Even though the protectivity of EVs has been proven in several *in vitro* and *in vivo* models the translation to humans will be a major challenge in the future. Unlike other approaches which failed to accomplish the translation from bench to bedside, the conserved mechanism of EV release and uptake in many species has the great potential that EVs might be of special use in the near future^65,73^.

## Conclusion

EVs are important mediators of cardiac protection and deliver specific molecules such as proteins and miRNAs to the recipient cells. The great majority of the investigated publications could proof the benefit of EVs especially by reducing cardiac damage or induction of angiogenesis. The inconsistency, in EV purification methods and experiments investigating EV mediated benefits, made it difficult to recapitulate data from different studies. Our evident conclusion is that EVs are important mediators of protection in cardiovascular diseases. These findings substantiate the assumption that EVs can serve as a potent therapeutic in the future. An urgent need, especially for a general EV-purification protocol, remains and has to be addressed in the future.

## Methods

The methods used in this review are in accordance with the recommendations provided by the Cochrane Collaboration^74^.

### Criteria for considering studies for this review

#### Types of studies

We included experimental research studies, which investigated EVs in *in vitro* or *in vivo* models (animal and human). We did not differentiate between exosomes, microvesicles or apoptotic bodies as long as the vesicular origin and effect matched our inclusion criteria.

#### Types of interventions

We included all studies, which investigated the protective effect of EVs. We did not further restrict type of intervention as long as the assumed protective effects of EVs or EV-derived components were the main focus of the study.

### Search methods for identification of studies

We identified trials through systematic searches of the following bibliographic databases on May 24^th^, 2018:

1. Cochrane Central Register of Controlled Trials in the Cochrane Library;
2. MEDLINE (Ovid, 1946 to May week 4, 2018);
3. Web of Science Core Collection (Thomson Reuters, 1900 to May 24^th^, 2018).

The following search strategy was applied to identify matching studies:

#1 extracellular vesicle* OR EV OR exosome* OR microvesicle*

#2 cardio OR cardiac OR heart OR cardioprotection

#3 protection OR *conditioning

#4 #1 AND #2 AND #3.

Reference lists of all primary studies and review articles were checked for additional references. We imported citations from each database into a reference management software (EndNote X8, PA, USA) and removed duplicates. Titles and abstracts of the selected articles were screened independently by two authors (SW, SK) and coded “suitable” or “not suitable”. In case of a disagreement a third author (CS) was questioned.

### Data extraction and quality assessment

Data from all suitable publications were reviewed, rated and extracted by two authors independently (SW, SK). In case of a disagreement a third author was questioned and the issue was discussed until the authors reached an agreement. The following information were extracted from every article: first author, year of publication, EV purification method, size of the detected EVs, damage model, mediator in EVs which transmitted the protectivity, assay to measure the beneficial effect of EVs and investigated EV marker. The EV purification methods were distinguished in methods based on ultracentrifugation, precipitation or others.

We checked the quality of the included studies by screening the purification methods, methodology to assess EV markers and the presence of electron microscopy (EM) pictures to visualize EV characteristics.

### Dealing with missing data

Investigators were contacted to obtain missing numerical data and to verify key study characteristics.

### Measures of treatment effect

We analysed dichotomous data as risk ratios (RR) with 95% confidence intervals (CI). For continuous data, we used the mean difference (MD) with 95% CI for outcomes measured in the same way between trials. We used the standardized mean difference (SMD) with 95% CI to combine data where the same outcome was measured but using different scales. Data reported as medians and interquartile ranges are merely described narratively, since they are presumably skewed distributed.

### Data synthesis

Statistical analyses were conducted with RevMan 5.3. Meta-analyses were only performed where appropriate. We made sure that the underlying question, EV source, and application as well as experimental setting were similar enough for pooling data. We used fixed-effects meta-analyses to produce a summary treatment effect across trials. We present the results as the treatment effect with its 95% confidence interval, and the estimates of T² and I².

### Reaching conclusion

Our conclusion is based on findings from the quantitative or narrative synthesis of included articles for this review. Suggestions are based on the intention to address uncertainties regarding EV purification as well as the performed experiments to investigate EV mediated protection to ensure a greater homogeneity in EV research to enable comparison across studies

## Electronic supplementary material

Study characteristics
