# Supplementary material for: Evaluation of the cardioprotective potential of extracellular vesicles – a systematic review and meta-analysis
Source: Sci Rep. 2018 Oct 24;8:15702. doi: 10.1038/s41598-018-33862-5 (PMC6200786; doi:10.1038/s41598-018-33862-5)
Supplement: Supplementary file 1 — Study characteristics [file 41598_2018_33862_MOESM1_ESM.docx]

Supplemental material for the following publication:

Evaluation of the cardioprotective potential of extracellular vesicles – a systematic review and meta-analysis

Sebastian Wendt^ab*^, Andreas Goetzenich^ab^, Claudia Goettsch^c^, Christian Stoppe^bd^, Christian Bleilevens^e^, Sandra Kraemer^ab+^ Carina Benstoem^bd+^

*^a^Department of Thoracic and Cardiovascular Surgery, University Hospital RWTH Aachen, Aachen, Germany*

*^b^Cardiovascular Critical Care & Anaesthesia research and evaluation (3CARE), University Hospital RWTH Aachen, Aachen, Germany*

^c^Department of Internal Medicine I-Cardiology, University Hospital RWTH Aachen, Aachen, Germany

^d^Department of Intensive Care Medicine, University Hospital RWTH Aachen, Aachen, Germany

^e^Department of Anaesthesiology, University Hospital RWTH Aachen, Aachen, Germany

^+^Equally contributed last authors

*Corresponding author: Sebastian Wendt, swendt@ukaachen.de, Department of Thoracic and Cardiovascular Surgery, University Hospital RWTH Aachen, Pauwelsstr. 30, 52074 Aachen, Germany Tel.: 0049 241-80-37715

(Arslan et al., 2013)

| EV purification procedure | Cell culture supernatant   - Centrifugation at 500 g - Filtration, 0.2 µm - Tangential flow filtration with 100 kDa - Chromatography (TSK Guard column SWXL, 6×40 mm and TSK gel G4000 SWXL, 7.8×300 mm, Tosoh Corp., Tokyo, Japan - 1^st^ peak concentrated with 100 kDa MWCO - Filtration, 0.2 µm |
| --- | --- |
| Electron microscopy for EVs | No |
| EV Marker | No |

(Balbi et al., 2017)

| EV purification procedure | Cell culture supernatant   - Centrifugation at 3,000 g for 20 min - Centrifugation at 10,000 g for 15 min - Centrifugation at 100,000 g for 90 min - Additional washing step at 100,000 g for 70 min |
| --- | --- |
| Electron microscopy for EVs | Yes   - Formvar coated copper grids - Fixation for 10 min with 2.5 % glutaraldehyde in PBS - Contrasted with 2 % uranyl acetate |
| EV Marker | TSG101, Alix, CD81, CD9, CD63, Annexin V |

(Bang et al., 2014)

| EV purification procedure | Cell culture supernatant   - Centrifugation at 500 g for 10 min - Centrifugation at 2,000g for 10 min - Centrifugation at 10,000 g for 30 min - Filtration, 0.22 µm - Centrifugation at 100,000 g for 70 min - Additional washing step at 100,000 g for 70 min |
| --- | --- |
| Electron microscopy for EVs | Yes   - Fixation in 2 % para formaldehyde (PFA) - Formvar carbon-coated grids - Washing and fixation with 1 % glutaraldehyde - Washing with water - Staining with 4 % uranyl-oxalate for 5 min - Embedding with 4 % uranyl acetate and 2 % methyl cellulose |
| EV Marker | CD63, GAPDH |

(Barile et al., 2014)

| EV purification procedure | Cell culture supernatant   - Centrifugation at 3,000 g for 15 min - Filtration, 0.22 µm - ExoQuick™ precipitation or - Ultracentrifugation at 100.000 g or - ExoSpin™ column precipitation |
| --- | --- |
| Electron microscopy for EVs | Yes   - fixation with 2.5 % glutaraldehyde - post fixation with 1% OsO_4_ with 1.5% K_4_Fe(CN)_6_ embedded in 1 % agar - processed according to the Epon812 procedure |
| EV Marker | CD63, CD9, CD81 |

(Shi et al., 2018)

| EV purification procedure | Cell culture supernatant   - Centrifugation at 500 g for 5 min - Centrifugation at 2,000 g for 30 min - ExoQuick-TC™ precipitation over night - Centrifugation at 1,500 g for 30 min - Centrifugation at 1,500 g for 5 min |
| --- | --- |
| Electron microscopy for EVs | Yes |
| EV Marker | CD9, CD63, HSP70 |

(Borosch et al., 2017)

| EV purification procedure | Cell culture supernatant   - Centrifugation at 300 g for10 min - Centrifugation at 2,000 g for15 min - Centrifugation at 12,000 g for 45 min - 0.22 µm filtration - Ultrafiltration - Size exclusion chromatography - Centrifugation at 118.000 g for 3 h |
| --- | --- |
| Electron microscopy for EVs | Yes   - EVs on formvar carbon coated grids 30 min - Fixed with 3 % glutaraldehyde - Washed 5 times - Negative staining with 4 % uranyl acetate in 2 % methyl cellulose for 10 min in the dark - Removing of excess liquid, air drying for 10 min |
| EV Marker | Alix, HSP70, CD63, Flot-1, CD81 |

(Chen et al., 2013)

| EV purification procedure | Cell culture supernatant   - Centrifugation at 1000 rpm for 10 min - Filtration, 0.22 µm - Precipitation with PEG over night at 4 °C - Centrifugation at 3000rpm for 30 min |
| --- | --- |
| Electron microscopy for EVs | Yes   - 3 µL of EVs on formvar carbon-coated 200-mesh copper electron microscopy grids - Uranyl acetate staining - Washing with PBS |
| EV Marker | CD63 |

(Cheow et al., 2016)

| EV purification procedure | Plasma   - Dilution with PBS - Centrifugation at 200 g for 30 min - Centrifugation at 2,000 g for 30 min - Centrifugation at 12,000 g for 60 min - Ultracentrifugation at 200,000 g for 18 h - Additional washing step at 200,000 g for 18 h - ExoQuick™ precipitation at 4°C over night |
| --- | --- |
| Electron microscopy for EVs | Yes   - Holey carbon filmed EM grids at 99 % humidity - Plunge into liquid ethane |
| EV Marker | CD9, CD81 |

(Davidson et al., 2018)

| EV purification procedure | Blood (rat), Cell culture supernatant   - Centrifugation at 1,600 g for 20 min - Centrifugation at 10,000 g for 30 min - Centrifugation at 100,000 g for 60 min - Additional washing step at 100,000 g for 60 min   Blood (human)   - Centrifugation at 1,600 g for 20 min - Centrifugation at 10,000 g for 30 min - Centrifugation at 100,000 g for 90 min - Additional washing step at 100,000 g for 60 min - Additional washing step at 100,000 g for 60 min |
| --- | --- |
| Electron microscopy for EVs | Yes   - Staining with 0.5 % uranyl acetate |
| EV Marker | CD81, HSP70 |

(de Couto et al., 2017)

| EV purification procedure | Cell culture supernatant   - Ultrafiltration - PEG precipitation over night - Centrifugation at 2,000 g for 30 min |
| --- | --- |
| Electron microscopy for EVs | No |
| EV Marker | CD63, Alix, HSP70 |

(T. Kang et al., 2016)

| EV purification procedure | Cell culture supernatant   - Centrifugation at 500 g for 10 min - Centrifugation at 12,000 g for 30 min - Centrifugation at 100,000 g for 60 min |
| --- | --- |
| Electron microscopy for EVs | Yes   - Fixation in 2.5 % glutaraldehyde and 1 % osmium tetroxide - Dehydration in alcohol - Coated with gold, sputter coating |
| EV Marker | Alix |

(Feng, Huang, Wani, Yu, & Ashraf, 2014)

| EV purification procedure | Cell culture supernatant   - Filtration, 0.3 µm - Centrifugation at 20,000 g for 20 min - Precipitation - Centrifugation at 100,000 g for 70 min - Centrifugation at 3,000 g for 15 min - ExoQuick™ precipitation - Centrifugation at 1,500 g for 30 min - Centrifugation at 1,500 g for 5 min |
| --- | --- |
| Electron microscopy for EVs | Yes |
| EV Marker | CD63 |

(Garcia, Ontoria-Oviedo, Gonzalez-King, Diez-Juan, & Sepulveda, 2015)

| EV purification procedure | Cell culture supernatant   - Centrifugation at 2,000 g for 10 min - Centrifugation at 10,000 g for 30 min - Filtration, 0.22 µm - Ultracentrifugation at 100,000 g for 70 min - Centrifugation at 2,000 g for 10 min - Centrifugation at 10,000 g for 30 min - Filtration, 0.22 µm - Ultracentrifugation at 100,000 g for 70 min - 30 % sucrose cushion, centrifugation at 100,000 g for 70 min - Additional washing step at 100,000 g for 70 min - ExoQuick TC™ |
| --- | --- |
| Electron microscopy for EVs | Yes   - Formvar carbon-coated grids - Contrasted with 2 % uranyl acetate |
| EV Marker | CD63, CD9, CD81 |

(Giricz et al., 2014)

| EV purification procedure | Coronary perfusates   - Dialyzation against 0.45 % saline containing 5mM EDTA for 4 h - Vaccum distillation to 40 ml - Filtration, 0.8 µm - Centrifugation at 12,200 g for 20 min - Filtration, 0.2 nm - Centrifugation at 100,000 g for 90 min |
| --- | --- |
| Electron microscopy for EVs | Yes   - Fixation with 4 % formaldehyde - Postfixation in OsO_4_ - Block-staining in 1 % uranyl acetate in 50 % EtOH - Dehydration in EtOh - Embedding in Taab 812 |
| EV Marker | HSP 60 |

(Gray et al., 2015)

| EV purification procedure | Cell culture supernatant   - Centrifugation at 10,000 g for 35 min - Centrifugation at 100,000 g for 70 min - 2 Additional washing steps at 100,000 g for 70 min |
| --- | --- |
| Electron microscopy for EVs | Yes   - Carbon-coated 200 mesh copper grid treated by glow discharge - 1 % phosphotungstic acid |
| EV Marker | CD9 |

(Gu et al., 2014)

| EV purification procedure | Cell culture supernatant   - Centrifugation at 1,000 g for 15 min - Ultracentrifugation at 100,000 g for 60 min |
| --- | --- |
| Electron microscopy for EVs | No |
| EV Marker | No |

(Ibrahim, Cheng, & Marban, 2014)

| EV purification procedure | Cell culture supernatant   - Centrifugation at 3,000 g for 15 min - ExoQuick™ precipitation |
| --- | --- |
| Electron microscopy for EVs | Yes |
| EV Marker | CD63 |

(K. Kang et al., 2015)

| EV purification procedure | Cell culture supernatant   - ExoQuick TC™ |
| --- | --- |
| Electron microscopy for EVs | Yes   - Fixation with 2 % glutaraldehyde and 2 % PFA in Sorensen’s phosphate buffer for 3 h - Fixation in 1 % OsO_4_ for 30 min - Dehydration in 50 %, 70 %, 80 %, 90 % and 100 % ethanol and embedding in Epon 812 - ≈60 nm section were cut with a ultramicrotome - Staining with uranyl acetate and Reynolds lead citrate |
| EV Marker | CD9, CD63 |

(Yu et al., 2015)

| EV purification procedure | Cell culture supernatant   - 0.22 µm filtration - Ultrafiltration - ExoQuick TC™ |
| --- | --- |
| Electron microscopy for EVs | Yes   - Fixation with 2 % PFA |
| EV Marker | CD9, CD63, HSP70 |

(Lai et al., 2010)

| EV purification procedure | Cell culture supernatant   - Concentration by tangential flow filtration - Ultrafiltration - Size exclusion fractionation by high-pressure liquid chromatography - Immunoprecipitation - Sucrose gradient centrifugation at 200,000 g for 16.6 h |
| --- | --- |
| Electron microscopy for EVs | Yes   - Pre-coating of formvar-coated grids with 0.01 % poly lysine for 1 min - Application of the sample - Drop of 1% pH 6 phosphotungstic acid |
| EV Marker | CD9, CD81, Alix |

(Li et al., 2014)

| EV purification procedure | Blood   - ExoQuick™ |
| --- | --- |
| Electron microscopy for EVs | Yes   - Fixation with glutaraldehyde on formvar-coated copper grids - Washing the grids twice with 0.1 M sodiumcacodylate and twice with water - Staining with 5 % uranyl acetate for 10 min |
| EV Marker | CD63 |

(Ma, Liu, Shen, Zhang, & Pan, 2015)

| EV purification procedure | Blood   - Centrifugation at 1,000 g for 15 min - Centrifugation at 15,000 g for 3 min - Centrifugation at 100,000 g for 1 h |
| --- | --- |
| Electron microscopy for EVs | Yes   - Carbon-coated holey film supported by a copper grid |
| EV Marker | CD41 for platelet derived particles |

(Namazi, Mohit, et al., 2018)

| EV purification procedure | Cell culture supernatant   - Centrifugation at 300 g for 10 min - Centrifugation at 2,500 g for 20 min - Centrifugation at 20,000 g for 25 min - Centrifugation at 100,000 g for 100 min - Additional washing step at 100,000 g for 100 min |
| --- | --- |
| Electron microscopy for EVs | Yes   - Drying on a glass substrate for 24 h |
| EV Marker | CD63, CD81 |

(Namazi, Namazi, et al., 2018)

| EV purification procedure | Cell culture supernatant   - Differential ultracentrifugation |
| --- | --- |
| Electron microscopy for EVs | Yes |
| EV Marker | CD63, CD81 |

(Obata et al., 2018)

| EV purification procedure | Cell culture supernatant   - Centrifugation at 800 g for 10 min - Centrifugation at 10,000 g for 30 min - Centrifugation at 110,000 g for 2 h - Additional washing step at 110,000 g for 2 h - EV fractionation by discontinuous iodixanol gradient   Blood   - Centrifugation at 10,000 g for 30 min - ExoQuick™ - Centrifugation at 1,500 g for 30 min - Centrifugation at 140,000 g for 2 h - Additional washing step at 140,000 g for 2 h - Centrifugation at 100,000 g for 18 h - Ultracentrifugation at 200,000 g for 2 h |
| --- | --- |
| Electron microscopy for EVs | Yes   - Fixation with 4 % PFA for 60 min - Incubation with primary antibody over night - Incubation with secondary antibody with gold particles for 2 h - Washing - Fixation with 2.5 % glutaraldehyde - Staining with 4 % uranyl acetate - Embedding in 1.8 % methylcellulose and 0.4 % uranyl acetate |
| EV Marker | Alix, HSP70, CD63, syntenin |

(Ong et al., 2014)

| EV purification procedure | Cell culture supernatant   - Centrifugation at 300 g for 10 min - Centrifugation at 2,000 g for 10 min - Centrifugation at 13,000 g for 15 min - 0.22 µm filtration - Ultrafiltration - Precipitation over night - Centrifugation at 12,000 g |
| --- | --- |
| Electron microscopy for EVs | Yes   - Fixation with 2 % formaldehyde - 300-meh formvar\|carbon coated grids - Fixation with 1 % glutaraldehyde - Contrasted and embedded |
| EV Marker | CD63, CD9 |

(Ribeiro-Rodrigues et al., 2017)

| EV purification procedure | Cell culture supernatant   - Differential centrifugation - 0.22 µm filtration - Centrifugation at 120,000 g for 70 min - Centrifugation at 100,000 g for 10 min with sucrose cushion - Additional washing step at 100,000 g for 70 min |
| --- | --- |
| Electron microscopy for EVs | Yes   - Fixation with 2 % PFA - Formvar-carbon coated grids - Washing and fixation with 1 % glutaraldehyde for 5 min - 8 Washing steps an contrasting with uranyl-oxalate solution for 5 min, Methyl-cellulose-uranyl for 10 min |
| EV Marker | Alix, HSP70, CD63, Flotillin-1, TSG-101, CD81, GAPDH |

(Svennerholm et al., 2016)

| EV purification procedure | Blood   - Centrifugation at 3,000 g for 30 min - Centrifugation at 10,000 g for 35 min - Centrifugation at 110,000 g for 2 h - Treatment with nuclease - Centrifugation at 110,000 g for 2 h on sucrose gradient - 2 Additional washing steps at 110,000 g for 2 h |
| --- | --- |
| Electron microscopy for EVs | (Yes) |
| EV Marker | (CD81) |

(Svennerholm et al., 2015)

| EV purification procedure | Blood   - Centrifugation at 3,000 g for 30 min - Centrifugation at 10,000 g for 35 min - Centrifugation at 110,000 g for 2 h - Treatment with nuclease - Centrifugation at 110,000 g for 2 h on sucrose gradient - 2 Additional washing steps at 110,000 g for 2 h |
| --- | --- |
| Electron microscopy for EVs | Yes |
| EV Marker | CD81 |

(Teng et al., 2015)

| EV purification procedure | Cell culture supernatant   - ExoQuick-TC™ |
| --- | --- |
| Electron microscopy for EVs | Yes |
| EV Marker | CD63 |

(Vandergriff et al., 2015)

| EV purification procedure | Cell culture supernatant   - 0.22 µm filtration - Ultrafiltration |
| --- | --- |
| Electron microscopy for EVs | No |
| EV Marker | CD63 |

(Vicencio et al., 2015)

| EV purification procedure | Blood (rat)   - Centrifugation at 1,600 g for 20 min - Centrifugation at 10,000 g for 30 min - Centrifugation twice at 100,000 g for 60 min   Blood (human)   - Centrifugation at 1,600 g for 20 min - Centrifugation at 10,000 g for 30 min - Centrifugation three times at 100,000 g for 60 min |
| --- | --- |
| Electron microscopy for EVs | Yes   - Staining with 0.5 % uranyl acetate - Immunostaining with cmHSP70.1 and appropriate gold-labelled secondary antibody - Post fixation and staining |
| EV Marker | CD63, CD81, HSP70 |

(Y. Wang et al., 2015)

| EV purification procedure | Cell culture supernatant   - Centrifugation at 1,000 rpm for 10 min - 0.22 µm filtration - PEG precipitation over night - Centrifugation at 3,000 rpm for 30 min |
| --- | --- |
| Electron microscopy for EVs | Yes   - Carbon-coated 200-mesh coper grids - Incubation for 5 min at room temperature - Uranyl acetate staining - Washing three times and semi drying |
| EV Marker | CD63, TSG101 |

(X. Wang et al., 2015)

| EV purification procedure | Cell culture supernatant   - Centrifugation at 3,000 rpm for 30 min - Centrifugation at 13,000 rpm for 30 min - 0.22 µm filtration - Centrifugation at 36,000 rpm for 3 h - Several washing steps - Centrifugation at 300 g for 10 min - Centrifugation at 1,200 g for 10 min twice, - Centrifugation at 10,000 g for 30 min - Centrifugation at 70,000 g for 60 min - Sucrose gradient - Centrifugation at 100,000 g for 16 h |
| --- | --- |
| Electron microscopy for EVs | No |
| EV Marker | CD81, CD63 |

(Wang et al., 2016)

| EV purification procedure | Cell culture supernatant   - Centrifugation at 3,000 rpm for 20 min - Centrifugation at 13,000 rpm for 30 min - 0.22 µm filtration - Centrifugation at 120,000 g for 2 h - Additional washing step   Blood   - Precipitation |
| --- | --- |
| Electron microscopy for EVs | No |
| EV Marker | CD63, CD81, HSP70 |

(Wider et al., 2018)

| EV purification procedure | Blood   - Centrifugation at 2,000 g - Centrifugation at 20,000 g for 45 min - 0.2 µm filtration - Centrifugation at 300,000 g for 12 h |
| --- | --- |
| Electron microscopy for EVs | No |
| EV Marker | Flot-1, HSP60 |

(Minghua et al., 2018)

| EV purification procedure | Blood   - Centrifugation at 1,500 g for 15 min - 0.22 µm filtration - Thrombin treatment - ExoQuick™ - Centrifugation at 1,500 g for 30 min |
| --- | --- |
| Electron microscopy for EVs | Yes   - Formvar carbon-coated 200-mesh copper grids - Uranyl acetate staining - Washing with PBS - Semi drying before imaging |
| EV Marker | CD63, CD81, CD9 |

(Xiao et al., 2016)

| EV purification procedure | Cell culture supernatant   - Centrifugation at 3,000 rpm for 15 min - 0.22 µm filtration - ExoQuickTC™ |
| --- | --- |
| Electron microscopy for EVs | Yes   - - Formvar carbon-coated 200-mesh copper grids - Incubation for 5 min - Uranyl acetate staining - Washing three times with PBS - Semi drying |
| EV Marker | Alix, CD63, CD9 |

(Yamaguchi et al., 2015)

| EV purification procedure | Blood   - Centrifugation at 2,000 g for 30 min - Centrifugation at 10,000 g for 30 min - Centrifugation at 10,000 g for 3 h - Washing step |
| --- | --- |
| Electron microscopy for EVs | No |
| EV Marker | CD9, HSP90 |

(Yu et al., 2013)

| EV purification procedure | Cell culture supernatant   - Centrifugation to remove cell debris - Ultrafiltration - ExoQuickTC™ |
| --- | --- |
| Electron microscopy for EVs | Yes |
| EV Marker | CD9, CD63, HSP70 |

(Z. Zhang et al., 2016)

| EV purification procedure | Cell culture supernatant   - Centrifugation at 3,000 g for 15 min - ExoQuickTC™ incubation over night - Centrifugation at 1,500 g for 30 min - Centrifugation at 1,500 g for 5 min |
| --- | --- |
| Electron microscopy for EVs | Yes |
| EV Marker | CD63 |

(X. Zhang et al., 2012)

| EV purification procedure | Cell culture supernatant   - ExoQuick™ |
| --- | --- |
| Electron microscopy for EVs | No |
| EV Marker | No |

(Zhao et al., 2015)

| EV purification procedure | Cell culture supernatant   - Centrifugation at 300 g for 20 min - Centrifugation at 2,000 g for 20 min - Centrifugation at 10,000 g for 30 min - Ultrafiltration - Centrifugation at 100,000 g for 2 h on sucrose cushion - Ultrafiltration - 0.22 µm filtration |
| --- | --- |
| Electron microscopy for EVs | Yes   - Formvar carbon-coated grid - Staining with phosphotungstic acid for 5 min |
| EV Marker | CD9, CD63 |

**Literature**

Arslan, F., Lai, R. C., Smeets, M. B., Akeroyd, L., Choo, A., Aguor, E. N., . . . de Kleijn, D. P. (2013). Mesenchymal stem cell-derived exosomes increase ATP levels, decrease oxidative stress and activate PI3K/Akt pathway to enhance myocardial viability and prevent adverse remodeling after myocardial ischemia/reperfusion injury. *Stem Cell Res, 10*(3), 301-312. doi:10.1016/j.scr.2013.01.002

Balbi, C., Piccoli, M., Barile, L., Papait, A., Armirotti, A., Principi, E., . . . Bollini, S. (2017). First Characterization of Human Amniotic Fluid Stem Cell Extracellular Vesicles as a Powerful Paracrine Tool Endowed with Regenerative Potential. *Stem Cells Transl Med, 6*(5), 1340-1355. doi:10.1002/sctm.16-0297

Bang, C., Batkai, S., Dangwal, S., Gupta, S. K., Foinquinos, A., Holzmann, A., . . . Thum, T. (2014). Cardiac fibroblast-derived microRNA passenger strand-enriched exosomes mediate cardiomyocyte hypertrophy. *J Clin Invest, 124*(5), 2136-2146. doi:10.1172/JCI70577

Barile, L., Lionetti, V., Cervio, E., Matteucci, M., Gherghiceanu, M., Popescu, L. M., . . . Vassalli, G. (2014). Extracellular vesicles from human cardiac progenitor cells inhibit cardiomyocyte apoptosis and improve cardiac function after myocardial infarction. *Cardiovasc Res, 103*(4), 530-541. doi:10.1093/cvr/cvu167

Borosch, S., Dahmen, E., Beckers, C., Stoppe, C., Buhl, E. M., Denecke, B., . . . Kraemer, S. (2017). Characterization of extracellular vesicles derived from cardiac cells in an in vitro model of preconditioning. *J Extracell Vesicles, 6*(1), 1390391. doi:10.1080/20013078.2017.1390391

Chen, L., Wang, Y., Pan, Y., Zhang, L., Shen, C., Qin, G., . . . Tang, Y. (2013). Cardiac progenitor-derived exosomes protect ischemic myocardium from acute ischemia/reperfusion injury. *Biochem Biophys Res Commun, 431*(3), 566-571. doi:10.1016/j.bbrc.2013.01.015

Cheow, E. S., Cheng, W. C., Lee, C. N., de Kleijn, D., Sorokin, V., & Sze, S. K. (2016). Plasma-derived Extracellular Vesicles Contain Predictive Biomarkers and Potential Therapeutic Targets for Myocardial Ischemic (MI) Injury. *Mol Cell Proteomics, 15*(8), 2628-2640. doi:10.1074/mcp.M115.055731

Davidson, S. M., Riquelme, J. A., Takov, K., Vicencio, J. M., Boi-Doku, C., Khoo, V., . . . Yellon, D. M. (2018). Cardioprotection mediated by exosomes is impaired in the setting of type II diabetes but can be rescued by the use of non-diabetic exosomes in vitro. *J Cell Mol Med, 22*(1), 141-151. doi:10.1111/jcmm.13302

de Couto, G., Gallet, R., Cambier, L., Jaghatspanyan, E., Makkar, N., Dawkins, J. F., . . . Marban, E. (2017). Exosomal MicroRNA Transfer Into Macrophages Mediates Cellular Postconditioning. *Circulation, 136*(2), 200-214. doi:10.1161/CIRCULATIONAHA.116.024590

Feng, Y., Huang, W., Wani, M., Yu, X., & Ashraf, M. (2014). Ischemic preconditioning potentiates the protective effect of stem cells through secretion of exosomes by targeting Mecp2 via miR-22. *PLoS One, 9*(2), e88685. doi:10.1371/journal.pone.0088685

Garcia, N. A., Ontoria-Oviedo, I., Gonzalez-King, H., Diez-Juan, A., & Sepulveda, P. (2015). Glucose Starvation in Cardiomyocytes Enhances Exosome Secretion and Promotes Angiogenesis in Endothelial Cells. *PLoS One, 10*(9), e0138849. doi:10.1371/journal.pone.0138849

Giricz, Z., Varga, Z. V., Baranyai, T., Sipos, P., Paloczi, K., Kittel, A., . . . Ferdinandy, P. (2014). Cardioprotection by remote ischemic preconditioning of the rat heart is mediated by extracellular vesicles. *J Mol Cell Cardiol, 68*, 75-78. doi:10.1016/j.yjmcc.2014.01.004

Gray, W. D., French, K. M., Ghosh-Choudhary, S., Maxwell, J. T., Brown, M. E., Platt, M. O., . . . Davis, M. E. (2015). Identification of therapeutic covariant microRNA clusters in hypoxia-treated cardiac progenitor cell exosomes using systems biology. *Circ Res, 116*(2), 255-263. doi:10.1161/CIRCRESAHA.116.304360

Gu, S., Zhang, W., Chen, J., Ma, R., Xiao, X., Ma, X., . . . Chen, Y. (2014). EPC-derived microvesicles protect cardiomyocytes from Ang II-induced hypertrophy and apoptosis. *PLoS One, 9*(1), e85396. doi:10.1371/journal.pone.0085396

Ibrahim, A. G., Cheng, K., & Marban, E. (2014). Exosomes as critical agents of cardiac regeneration triggered by cell therapy. *Stem Cell Reports, 2*(5), 606-619. doi:10.1016/j.stemcr.2014.04.006

Kang, K., Ma, R., Cai, W., Huang, W., Paul, C., Liang, J., . . . Wang, Y. (2015). Exosomes Secreted from CXCR4 Overexpressing Mesenchymal Stem Cells Promote Cardioprotection via Akt Signaling Pathway following Myocardial Infarction. *Stem Cells Int, 2015*, 659890. doi:10.1155/2015/659890

Kang, T., Jones, T. M., Naddell, C., Bacanamwo, M., Calvert, J. W., Thompson, W. E., . . . Liu, D. (2016). Adipose-Derived Stem Cells Induce Angiogenesis via Microvesicle Transport of miRNA-31. *Stem Cells Transl Med, 5*(4), 440-450. doi:10.5966/sctm.2015-0177

Lai, R. C., Arslan, F., Lee, M. M., Sze, N. S., Choo, A., Chen, T. S., . . . Lim, S. K. (2010). Exosome secreted by MSC reduces myocardial ischemia/reperfusion injury. *Stem Cell Res, 4*(3), 214-222. doi:10.1016/j.scr.2009.12.003

Li, J., Rohailla, S., Gelber, N., Rutka, J., Sabah, N., Gladstone, R. A., . . . Redington, A. N. (2014). MicroRNA-144 is a circulating effector of remote ischemic preconditioning. *Basic Res Cardiol, 109*(5), 423. doi:10.1007/s00395-014-0423-z

Ma, F., Liu, H., Shen, Y., Zhang, Y., & Pan, S. (2015). Platelet-derived microvesicles are involved in cardio-protective effects of remote preconditioning. *Int J Clin Exp Pathol, 8*(9), 10832-10839.

Minghua, W., Zhijian, G., Chahua, H., Qiang, L., Minxuan, X., Luqiao, W., . . . Xiaoshu, C. (2018). Plasma exosomes induced by remote ischaemic preconditioning attenuate myocardial ischaemia/reperfusion injury by transferring miR-24. *Cell Death Dis, 9*(3), 320. doi:10.1038/s41419-018-0274-x

Namazi, H., Mohit, E., Namazi, I., Rajabi, S., Samadian, A., Hajizadeh-Saffar, E., . . . Baharvand, H. (2018). Exosomes secreted by hypoxic cardiosphere-derived cells enhance tube formation and increase pro-angiogenic miRNA. *J Cell Biochem, 119*(5), 4150-4160. doi:10.1002/jcb.26621

Namazi, H., Namazi, I., Ghiasi, P., Ansari, H., Rajabi, S., Hajizadeh-Saffar, E., . . . Mohit, E. (2018). Exosomes Secreted by Normoxic and Hypoxic Cardiosphere-derived Cells Have Anti-apoptotic Effect. *Iran J Pharm Res, 17*(1), 377-385.

Obata, Y., Kita, S., Koyama, Y., Fukuda, S., Takeda, H., Takahashi, M., . . . Shimomura, I. (2018). Adiponectin/T-cadherin system enhances exosome biogenesis and decreases cellular ceramides by exosomal release. *JCI Insight, 3*(8). doi:10.1172/jci.insight.99680

Ong, S. G., Lee, W. H., Huang, M., Dey, D., Kodo, K., Sanchez-Freire, V., . . . Wu, J. C. (2014). Cross talk of combined gene and cell therapy in ischemic heart disease: role of exosomal microRNA transfer. *Circulation, 130*(11 Suppl 1), S60-69. doi:10.1161/CIRCULATIONAHA.113.007917

Ribeiro-Rodrigues, T. M., Laundos, T. L., Pereira-Carvalho, R., Batista-Almeida, D., Pereira, R., Coelho-Santos, V., . . . Girao, H. (2017). Exosomes secreted by cardiomyocytes subjected to ischaemia promote cardiac angiogenesis. *Cardiovasc Res, 113*(11), 1338-1350. doi:10.1093/cvr/cvx118

Shi, B., Wang, Y., Zhao, R., Long, X., Deng, W., & Wang, Z. (2018). Bone marrow mesenchymal stem cell-derived exosomal miR-21 protects C-kit+ cardiac stem cells from oxidative injury through the PTEN/PI3K/Akt axis. *PLoS One, 13*(2), e0191616. doi:10.1371/journal.pone.0191616

Svennerholm, K., Rodsand, P., Hellman, U., Lundholm, M., Waldenstrom, A., Biber, B., . . . Haney, M. (2015). Myocardial ischemic preconditioning in a porcine model leads to rapid changes in cardiac extracellular vesicle messenger RNA content. *Int J Cardiol Heart Vasc, 8*, 62-67. doi:10.1016/j.ijcha.2015.05.006

Svennerholm, K., Rodsand, P., Hellman, U., Waldenstrom, A., Lundholm, M., Ahren, D., . . . Haney, M. (2016). DNA Content in Extracellular Vesicles Isolated from Porcine Coronary Venous Blood Directly after Myocardial Ischemic Preconditioning. *PLoS One, 11*(7), e0159105. doi:10.1371/journal.pone.0159105

Teng, X., Chen, L., Chen, W., Yang, J., Yang, Z., & Shen, Z. (2015). Mesenchymal Stem Cell-Derived Exosomes Improve the Microenvironment of Infarcted Myocardium Contributing to Angiogenesis and Anti-Inflammation. *Cell Physiol Biochem, 37*(6), 2415-2424. doi:10.1159/000438594

Vandergriff, A. C., de Andrade, J. B., Tang, J., Hensley, M. T., Piedrahita, J. A., Caranasos, T. G., & Cheng, K. (2015). Intravenous Cardiac Stem Cell-Derived Exosomes Ameliorate Cardiac Dysfunction in Doxorubicin Induced Dilated Cardiomyopathy. *Stem Cells Int, 2015*, 960926. doi:10.1155/2015/960926

Vicencio, J. M., Yellon, D. M., Sivaraman, V., Das, D., Boi-Doku, C., Arjun, S., . . . Davidson, S. M. (2015). Plasma exosomes protect the myocardium from ischemia-reperfusion injury. *J Am Coll Cardiol, 65*(15), 1525-1536. doi:10.1016/j.jacc.2015.02.026

Wang, X., Gu, H., Huang, W., Peng, J., Li, Y., Yang, L., . . . Fan, G. C. (2016). Hsp20-Mediated Activation of Exosome Biogenesis in Cardiomyocytes Improves Cardiac Function and Angiogenesis in Diabetic Mice. *Diabetes, 65*(10), 3111-3128. doi:10.2337/db15-1563

Wang, X., Gu, H., Qin, D., Yang, L., Huang, W., Essandoh, K., . . . Fan, G. C. (2015). Exosomal miR-223 Contributes to Mesenchymal Stem Cell-Elicited Cardioprotection in Polymicrobial Sepsis. *Sci Rep, 5*, 13721. doi:10.1038/srep13721

Wang, Y., Zhang, L., Li, Y., Chen, L., Wang, X., Guo, W., . . . Tang, Y. (2015). Exosomes/microvesicles from induced pluripotent stem cells deliver cardioprotective miRNAs and prevent cardiomyocyte apoptosis in the ischemic myocardium. *Int J Cardiol, 192*, 61-69. doi:10.1016/j.ijcard.2015.05.020

Wider, J., Undyala, V. V. R., Whittaker, P., Woods, J., Chen, X., & Przyklenk, K. (2018). Remote ischemic preconditioning fails to reduce infarct size in the Zucker fatty rat model of type-2 diabetes: role of defective humoral communication. *Basic Res Cardiol, 113*(3), 16. doi:10.1007/s00395-018-0674-1

Xiao, J., Pan, Y., Li, X. H., Yang, X. Y., Feng, Y. L., Tan, H. H., . . . Yu, X. Y. (2016). Cardiac progenitor cell-derived exosomes prevent cardiomyocytes apoptosis through exosomal miR-21 by targeting PDCD4. *Cell Death Dis, 7*(6), e2277. doi:10.1038/cddis.2016.181

Yamaguchi, T., Izumi, Y., Nakamura, Y., Yamazaki, T., Shiota, M., Sano, S., . . . Iwao, H. (2015). Repeated remote ischemic conditioning attenuates left ventricular remodeling via exosome-mediated intercellular communication on chronic heart failure after myocardial infarction. *Int J Cardiol, 178*, 239-246. doi:10.1016/j.ijcard.2014.10.144

Yu, B., Gong, M., Wang, Y., Millard, R. W., Pasha, Z., Yang, Y., . . . Xu, M. (2013). Cardiomyocyte protection by GATA-4 gene engineered mesenchymal stem cells is partially mediated by translocation of miR-221 in microvesicles. *PLoS One, 8*(8), e73304. doi:10.1371/journal.pone.0073304

Yu, B., Kim, H. W., Gong, M., Wang, J., Millard, R. W., Wang, Y., . . . Xu, M. (2015). Exosomes secreted from GATA-4 overexpressing mesenchymal stem cells serve as a reservoir of anti-apoptotic microRNAs for cardioprotection. *Int J Cardiol, 182*, 349-360. doi:10.1016/j.ijcard.2014.12.043

Zhang, X., Wang, X., Zhu, H., Kranias, E. G., Tang, Y., Peng, T., . . . Fan, G. C. (2012). Hsp20 functions as a novel cardiokine in promoting angiogenesis via activation of VEGFR2. *PLoS One, 7*(3), e32765. doi:10.1371/journal.pone.0032765

Zhang, Z., Yang, J., Yan, W., Li, Y., Shen, Z., & Asahara, T. (2016). Pretreatment of Cardiac Stem Cells With Exosomes Derived From Mesenchymal Stem Cells Enhances Myocardial Repair. *J Am Heart Assoc, 5*(1). doi:10.1161/JAHA.115.002856

Zhao, Y., Sun, X., Cao, W., Ma, J., Sun, L., Qian, H., . . . Xu, W. (2015). Exosomes Derived from Human Umbilical Cord Mesenchymal Stem Cells Relieve Acute Myocardial Ischemic Injury. *Stem Cells Int, 2015*, 761643. doi:10.1155/2015/761643
